# Prolonged Release and Functionality of Interleukin-10 Encapsulated within PLA-PEG Nanoparticles

**DOI:** 10.3390/nano9081074

**Published:** 2019-07-26

**Authors:** Skyla A. Duncan, Saurabh Dixit, Rajnish Sahu, David Martin, Dieudonné R. Baganizi, Elijah Nyairo, Francois Villinger, Shree R. Singh, Vida A. Dennis

**Affiliations:** 1 Center for NanoBiotechnology & Life Sciences Research, Department of Biological Sciences, Alabama State University, 915 South Jackson Street, Montgomery, AL 36104, USA; 2 New Iberia Research Center, University of Louisiana at Lafayette, 4401 W Admiral Doyle Drive, New Iberia, LA 70560, USA

**Keywords:** chlamydia, recombinant major outer membrane protein (rMOMP), PLA-PEG nanoparticles, IL-10, cytokines, SOCS1, SOCS3, inflammation, anti-inflammatory, therapy

## Abstract

Inflammation, as induced by the presence of cytokines and chemokines, is an integral part of chlamydial infections. The anti-inflammatory cytokine, interleukin (IL)-10, has been reported to efficiently suppress the secretion of inflammatory cytokines triggered by *Chlamydia* in mouse macrophages. Though IL-10 is employed in clinical applications, its therapeutic usage is limited due to its short half-life. Here, we document the successful encapsulation of IL-10 within the biodegradable polymeric nanoparticles of PLA-PEG (Poly (lactic acid)-Poly (ethylene glycol), to prolong its half-life. Our results show the encapsulated-IL-10 size (~238 nm), zeta potential (−14.2 mV), polydispersity index (0.256), encapsulation efficiency (~77%), and a prolonged slow release pattern up to 60 days. Temperature stability of encapsulated-IL-10 was favorable, demonstrating a heat capacity of up to 89 °C as shown by differential scanning calorimetry analysis. Encapsulated-IL-10 modulated the release of IL-6 and IL-12p40 in stimulated macrophages in a time- and concentration-dependent fashion, and differentially induced SOCS1 and SOCS3 as induced by chlamydial stimulants in macrophages. Our finding offers the tremendous potential for encapsulated-IL-10 not only for chlamydial inflammatory diseases but also biomedical therapeutic applications.

## 1. Introduction

*Chlamydia trachomatis* is the etiological agent responsible for one of the most prevalent sexually transmitted infections, that, if left untreated, manifests with potentially serious reproductive consequences, including salpingitis, endometritis, pelvic inflammatory disease (PID), ectopic pregnancy, and tubal factor infertility in women [1]. Genital chlamydial infections are also associated with an increased risk of the transmission or acquisition of HIV and are also a risk factor for the development of cervical carcinoma, making them a major public health concern [1]. Whilst the early diagnosis and treatment of infected individuals is required to prevent the spread of the disease and severe sequelae [1], the complexities of the asymptomatic nature of the disease, possibility of reinfection due to the failure of initial protective immune memory, and lack of an effective vaccine have limited the efficacy of treatment options that are currently available [2]. 

During a *Chlamydia*-induced inflammatory response, the presence of damaged cells activates macrophages to release a series of pro-inflammatory mediators (IL-1, IL-6, TNF-α, etc.) that contribute to the chronic inflammation associated with the disease [3]. Our research group has implicated Interleukin-10 (IL-10), an anti-inflammatory cytokine, in effectively modulating acute inflammatory responses elicited by live and UV inactivated *C. trachomatis* [4] and its major outer membrane protein (MOMP) moiety. IL-10 is an immuno-regulatory Th2 cytokine produced by many immune cells, including T cells, B cells, natural killer cells, antigen-presenting cells, mast cells, and granulocytes that play a central role in infection by limiting the immune response to pathogens and thereby preventing damage to the host [5,6]. Consequently, IL-10 has been investigated as a therapeutic agent for many autoimmune and inflammatory diseases [7,8,9,10,11,12,13,14], including our recent studies involving *C. trachomatis* [4]. Interestingly, clinical trials involving subcutaneous IL-10 administration to psoriatic patients for 3–7 weeks proved to be an efficient immunosuppressant and a well-tolerated treatment in decreasing skin lesions, with long-term IL-10 therapy even decreasing the incidence of relapse and extending the disease-free interval [15,16]. However, the short biological half-life of IL-10 limited its usage and required large, and frequent, dosage administration for prolonged anti-psoriatic activity, making the therapy somewhat restricted [16]. Henceforth, it is necessary to explore potential delivery systems, such as the novel use of biodegradable nanoparticles, which can ultimately extend the biological half-life of IL-10 and promote optimal anti-psoriatic/anti-inflammatory activity. 

Biodegradable polymers have typically been frequently used as drug delivery vehicles due to their bioavailability, better encapsulation, control release, and less toxic properties [17]. Poly-(lactic acid) (PLA) is the most widely used polymer in drug delivery systems and has been approved by the FDA for clinical use. However, the hydrophobicity of PLA raises concerns for further biological and biomedical applications. Polyethylene glycol (PEG) is the most popular hydrophilic polymer for the surface modification of nanoparticulate drug carriers and has been used to modify the hydrophobic PLA to form the amphiphilic copolymer PLA-PEG. Specifically, we chose to encapsulate IL-10 in PLA-PEG polymers because, together, they are extensively used to develop nano-encapsulating therapeutic materials for targeted delivery potential, low toxicity, and controlled release applications due to their biocompatibility and bioadhesive properties [18,19]. The hydrophobic PLA core can encapsulate our anti-inflammatory agent IL-10 and ensure reproducible release kinetics, while the PEG layer provides the nanoparticle with stealthy properties [20]. This copolymer combination has also been shown to enhance the therapeutic activity of encapsulated drugs [21] and consequently, is more commonly proposed for proficient drug delivery. Therefore, our PEGylated-PLA IL-10 nanoparticle approach provides great promise for extended immunotherapeutic effects for the treatment of genital *Chlamydia*.

In this present study, we explored the hypothesis that encapsulated IL-10 will maintain a slow release of functional IL-10. First, by in vitro studies, we determined the physiochemical characteristics of encapsulated-IL-10 (size, absorbance, thermal stability, and chemical compositions), encapsulation efficiency, and release pattern. Next, we compared bare and encapsulated IL-10-mediated inhibition of *Chlamydial* inflammatory responses in mouse J774 macrophages, and the ensuing effect on the mRNA transcriptional expression of the suppressor of cytokine signaling (SOCS)1 and SOCS3. Herein, we present and discuss our findings in the context of PLA-PEG nanoparticles as an effective delivery system not only for IL-10 anti-inflammatory effects in chlamydial stimulated macrophages, but also for usage in biomedical applications.

## 2. Materials and Methods

### 2.1. Cell Line

Mouse J774 macrophages were obtained from the American Type Culture Collection (ATCC, Manassas, VA, USA). They were cultured in Dulbecco Modified Eagle Medium (DMEM) (ATCC) supplemented with 10% heat-inactivated fetal bovine serum (FBS) (Invitrogen, Carlsbad, CA, USA) and 1 µg/mL antibiotic and antimycotic (Invitrogen) (complete medium) [22]. Cells were maintained at 37 °C in a humidified incubator containing 5% CO_2_ for various periods of time, depending on the experimental procedure.

### 2.2. Stimulants

The *Chlamydia muridarum* [strain Nigg II; previously called *C. trachomatis* mouse pneumonitis (MoPn) biovar] recombinant major outer membrane protein (rMOMP) was cloned as previously reported [23]. *Escherichia coli* lipopolysaccharide (LPS) was incubated with macrophages in complete media at a concentration of 1 µg/mL to serve as a positive control for our preliminary experiments. Mouse recombinant IL-10 was purchased from BD Biosciences (San Jose, CA, USA). 

The recombinant monkey IL-10 was cloned and expressed as an Ig-Fc fusion protein with the Fc mutated to abolish binding to FcyR and complemented by the Resource for Nonhuman Primate Immune Reagents (NIH R24OD010947) at the New Iberia Research Center (University of Louisiana at Lafayette). The protein expression was conducted in S-2 drosophila cells cultured in suspension and induced by copper sulfate. After purification with protein G-Sepharose, the cytokine was dialyzed to bicarbonate buffer prior to lyophilization. The absence of endotoxin was verified by the Limulus amebocyte lysis assay, and bioactivity was measured by the inhibition of IFN-γ production by Concanavalin A-stimulated rhesus peripheral mononuclear cells.

### 2.3. Stimulation of Macrophages

Mouse J774 macrophages (3 × 10^6^ cells/mL) were stimulated with LPS (1 μg/mL) or rMOMP (10 μg/mL) in the presence and absence of mouse IL-10 (1 or 10 ng/mL) and monkey IL-10 (1, 10, 100. or 1000 ng/mL) to assess the effect of both IL-10 species on the expression of cytokines, as induced by rMOMP of *Chlamydia*. Mouse J774 macrophages (3 × 10^6^ cells/mL) were also stimulated with rMOMP (10 μg/mL) in the presence and absence of mouse IL-10 (10 ng/mL) for 24 h to assess the effect of IL-10 on the time-dependent induction of suppressor of cytokine signaling (SOCS)1 and SOCS3. Stimulated macrophage cultures were incubated at 37 °C with 5% CO_2_ for various time-periods, depending on the specific experiment. Post-stimulation, cell-free supernatants were collected by centrifugation at 450× *g* for 10 min at 4 °C and stored at −80 °C until used for cytokine ELISAs. Cell pellets were used for RNA extraction and subsequent TaqMan quantitative real time-polymerase chain reaction (qRT-PCR) analysis. as described below.

### 2.4. Preparation of Nanoparticles

Monkey recombinant IL-10 was encapsulated in PEG-b-PLA Diblock polymer nanoparticles by a modified water/oil/water double emulsion evaporation technique essentially as described previously [24,25]. Briefly, 300 mg of PLA-PEG was emulsified in Ethyl acetate, followed by the addition of 1 mg of monkey recombinant IL-10, homogenization, and the addition of 1% Polyvinyl Alcohol (PVA). The resulting double emulsion was gently stirred overnight at room temperature (RT) to evaporate the organic solvents, harvested by ultracentrifugation, washed, and lyophilized in the presence of 5% trehalose (used as a stabilizer) to obtain encapsulated-IL-10 (PLA-PEG-IL-10). Sterile Phosphate buffered saline (PBS) was used in the primary emulsion formation to prepare PLA-PEG-PBS nanoparticles to serve as a negative control. All lyophilized nanoparticles were stored at −80 °C in a sealed container until used.

### 2.5. Encapsulation Efficiency

The encapsulation efficiency of encapsulated IL-10 was studied as described previously [24,25]. Briefly, lyophilized encapsulated IL-10 (20 mg) was added to 1 mL of 0.1 N NaOH containing 2% sodium dodecyl sulfate (SDS), and shaken overnight at RT; the supernatant was collected by centrifugation at 13,680× *g* for 5 min and then stored at −20 °C. The Micro bicinchoninic acid assay (BCA) protein assay was used to quantify free IL-10 in supernatants, and the absorbance was read at 570 nm using a microplate reader (TECAN US Inc., Durham, NC, USA). Background readings were corrected by subtracting the optical density (OD) values of supernatants from the encapsulated PBS negative control nanoparticles. The IL-10 encapsulation efficiency (*EE*) was calculated using the formula below, where *A* is the total IL-10 amount and *B* is the free IL-10 amount. These measurements were performed three times. 

$$EE\;\left(\%\right)=\left[\frac{A-B}{A}\right]100$$

### 2.6. Zeta-Sizer, Zeta-Potential, and Polydispersity Index (PDI) Measurements 

The sizes of PLA-PEG-IL-10 and PLA-PEG-PBS nanoparticles were measured by dynamic light scattering employing a zeta-sizer Nano-ZS instrument (Malvern Instruments, UK), as described previously [22,26]. Briefly, nanoparticles were suspended in filtered distilled water, sonicated, and placed in a disposable cuvette for size, zeta potential, and PDI measurements. Each sample was measured three times and is reported as the mean of triplicates for the zeta-sizer (diameter in nanometers), zeta-potential (millivolt), and PDI. 

### 2.7. UV Visible Spectra (UV-Vis)

Encapsulated nanoparticles (5 mg each) and recombinant IL-10 (1 mg/mL) for spectroscopy were prepared by dissolving them in DNAse-RNAse free water, and the UV visible spectra were taken using the Beckman Coulter DU 800 UV/Vis spectrophotometer (Brea, CA, USA) [25].

### 2.8. Fourier Transform-Infrared Spectrometry (FT-IR) 

FT-IR spectra were recorded for encapsulated nanoparticles in attenuated total reflectance (ATR) mode using an IR spectrophotometer Nicolet 380 (Thermo Fisher, Waltham, MA, USA) [25]. The spectra were obtained with 64 scans/sample ranging from 4000 to 1000 cm^−1^ and a resolution of 4 cm^−1^. The sample chamber was purged with dry N_2_ gas.

### 2.9. Differential Scanning Calorimetry (DSC)

Temperature stability experiments were carried out using DSC (Toledo DSC822e; Mettler, Columbus, OH, USA), as described previously [22]. Encapsulated nanoparticles (10 mg each) were heated at a rate of 20 °C per min from 30 to 120 °C under nitrogen and then cooled from 120 to 30 °C at the same rate.

### 2.10. In Vitro Release of Encapsulated-IL-10 

The release of IL-10 from the encapsulated IL-10 was determined as described previously [23,25]. Encapsulated nanoparticles (3.6 mg containing 64 µg of encapsulated IL-10) were suspended in PBS containing 0.01% sodium azide (500 μL) and incubated at 37 °C. At various time-intervals (1, 2, and 4 h, and days 1–60), supernatants were collected by centrifugation at 13,680× *g* for 5 min and stored at −20 °C until assayed. The released IL-10 was measured using the Micro BCA assay, as calculated from the standard curve. Absorbance reading of the encapsulated PBS control nanoparticles was subtracted from those of the encapsulated IL-10, and the results are expressed as an accumulative release over the entire 60-day assessment period. 

### 2.11. In Vitro Stimulation of Macrophages with Encapsulated Nanoparticles 

The anti-inflammatory effect of the PLA-PEG-IL-10 nanoparticles was investigated by exposing nanoparticles to mouse J774 macrophages in the presence and absence of rMOMP. Macrophages (3 × 10^6^ cells/mL) were cultured in 24-well plates and media containing encapsulated nanoparticles at various concentrations (0–100 ng/mL) in the presence and absence of rMOMP (10 μg/mL) was added, followed by incubation for an additional 24–72 h. Unstimulated cells and PLA-PEG-PBS served as negative controls. Cell-free supernatants were collected by centrifugation and stored at −20 °C until used.

### 2.12. Cytokines Measurement 

Cytokine enzyme-linked immunosorbent assays (ELISAs) were used to quantify concentrations of mouse IL-6 and IL-12p40 in cell-free supernatants using BD Biosciences (San Jose, CA, USA) Opti-EIA kits [24,25]. 

### 2.13. RNA Extraction and Quantitative Real Time-PCR (qRT-PCR)

Total RNA was isolated from unstimulated and stimulated cells using the Qiagen RNeasy mini plus Kit (Qiagen Inc., Valencia, CA, USA), which included a DNase-I digestion step or the use of gDNA eliminator columns. The resulting RNA samples were transcribed into cDNA using the High Capacity cDNA Reverse Transcription Kit (Applied Biosystems, Foster city, CA, USA). Next, TaqMan qRT-PCR was employed as described previously [25,27] to assess the mRNA gene transcripts of the following genes: socs1 [Mm00782550_s1], socs3 [Mm00545913_s1], stat1 [Mm01219775_m1], and stat3 [Mm01219775_m1], using TaqMan^®^ gene expression assays (Applied Biosystems, Foster city, CA, USA) as reported previously [28,29]. Amplification of gene transcripts was performed according to the manufacturer’s protocol using ABI ViiA™ 7 real-time PCR (Applied Biosystems) and standard amplification conditions. The relative changes in gene expression were calculated using the equation 2−ΔΔCT, where all values were normalized with respect to the “housekeeping” gene GAPDH (glyceraldehyde 3-phosphate dehydrogenase) [Mm99999915_g1] mRNA levels. Amplification using 50 ng RNA was performed in a total volume of 20 μL. Each real-time PCR assay was performed in triplicate and repeated at least three times. 

### 2.14. Statistical Analysis 

Data are expressed as the mean ± SD of samples run in triplicate, and each experiment was repeated at least three to four different times. Statistical analyses were performed using one- or two-way analysis of variance (ANOVA), followed by Tukey’s Post-test using GraphPad Prism 6 Software (San Diego, CA, USA). Statistical significance was established and *p* values < 0.05 were considered statistically significant (* *p* < 0.05; ** *p* < 0.01, *** *p* < 0.001, and **** *p* < 0.0001). 

## 3. Results

### 3.1. Monkey IL-10 Is Efficacious at Suppressing Chlamydial-Induced Inflammatory Cytokines

LPS is a major component of the cell wall of Gram-negative bacteria [30], which triggers the release of cytokines, including IL-6 [31] and IL-12 p40 [32], from macrophages. MOMP is also the most abundant protein of *Chlamydia* and is highly immunogenic [33]. Because LPS and rMOMP can induce inflammation, we determined the anti-inflammatory effect of monkey IL-10 on cytokines, as induced by LPS and chlamydial rMOMP in mouse macrophages. As demonstrated in Supplementary Data Figure S1, all doses of mouse IL-10 significantly reduced LPS- and rMOMP-induced IL-6 production, being maximal at the 10 ng/mL dosage. Recombinant Monkey IL-10, on the other hand, dose-dependently inhibited IL-6 levels (*p* < 0.0001) in mouse macrophages, but at the higher concentrations of 100 and 1000 ng/mL, suggesting species specificity for exerting their anti-inflammatory effects. Nonetheless, this data shows that monkey IL-10 is functional by inhibiting pro-inflammatory responses, as elicited by rMOMP in mouse macrophages, and serves as validation for its use in the encapsulation studies described below. 

### 3.2. Chlamydia rMOMP Triggers Enhanced mRNA Gene Transcripts of SOCS3 and STAT1 Alone or Combined with Mouse IL-10 in Macrophages

Suppressors of cytokine signaling (SOCS) have been implicated in many inflammatory diseases for their critical role as negative regulators of cytokine signaling by inhibiting the JAK/STAT pathway [34,35]. Moreover, they have been implicated as potential mediators of the IL-10 modulation of inflammatory responses in macrophages [35,36,37]. As such, initial experiments were conducted in an attempt to delineate a correlative link between the induction of SOCS1 and SOCS3 with the capacity of IL-10 to modulate chlamydial-induced inflammatory responses by first investigating whether or not rMOMP can activate mediators of the JAK/STAT pathway. Mouse J774 macrophages were stimulated with LPS (1 µg/mL) or rMOMP (10 µg/mL) for 24 h to quantify, by TaqMan qRT-PCR, the mRNA gene transcripts of SOCS1 and SOCS3, along with the transcription factors, STAT1 and STAT3. Our results, as depicted in Supplementary Data Figure S2, disclosed the induction of SOCS1 and SOCS3 expression, in response to the rMOMP stimulation of macrophages. The SOCS3 expression level was approximately four-fold higher than that of SOCS1, suggesting its inflammatory potential and possibly regulating its induced inflammation. We also disclosed up to a five-fold higher expression of STAT1 than STAT3, as induced by rMOMP in macrophages, which suggests the capacity of *Chlamydia* to control early innate immune responses. Similar results were obtained for LPS.

### 3.3. Physical-Structural Characterization of Monkey Recombinant IL-10 Encapsulated in PLA-PEG Nanoparticles

**Figure 1:**
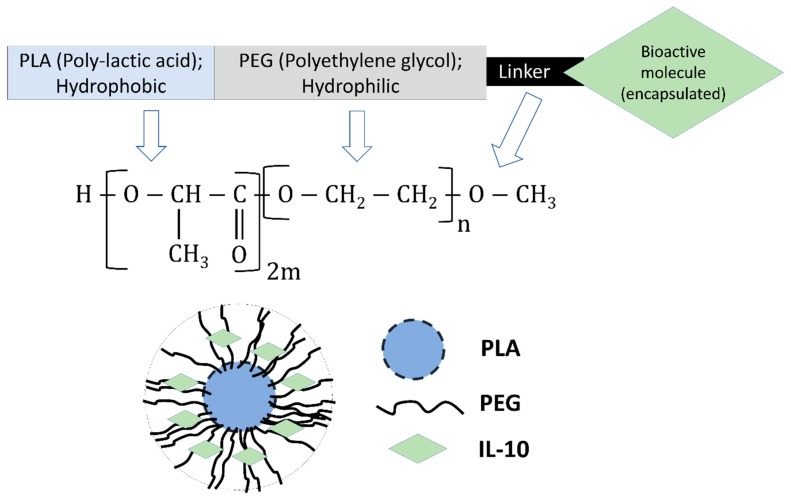
Schematic representation of Interleukin-10 (IL-10) encapsulated in Poly-(lactic acid)-Polyethylene glycol (PLA-PEG) nanoparticles.

Given that our ultimate goal is to prolong the biological half-life of IL-10 by its encapsulation within biodegradable nanoparticles, we selected employing recombinant monkey IL-10 for encapsulation due to the following reasons: (1) purchase of large amounts of mouse recombinant is costly; (2) monkey recombinant IL-10 is functional in mouse macrophages. For Supplementary Data Figures S1 and S2, large quantities of recombinant monkey IL-10 were available for the encapsulation studies. Our selection of PLA-PEG for the encapsulation of IL-10 (Figure 1) is due to its targeted delivery potential, low toxicity, and controlled release applications due to its biocompatibility and bioadhesive properties [18,19].

#### 3.3.1. Encapsulation Efficiency, Zeta-Sizing, Zeta-Potential, and PDI

A schematic of the encapsulation of IL-10 in PLA-PEG nanoparticles is depicted in Figure 1. We quantified the encapsulation efficiency of the encapsulated IL-10, since the quantity of encapsulated IL-10 in the nanoparticles is an important factor for determining the release profile of a drug delivery system. A modified water/oil/water double emulsion method was employed for the fabrication of encapsulated-IL-10, resulting in a ~77% encapsulation efficiency (Table 1). 

**Figure 2:**
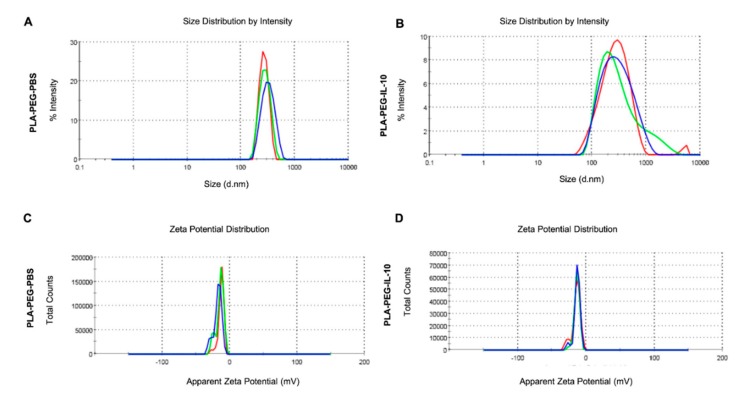
Zeta-sizing and zeta-potential measurements of encapsulated Interleukin-10 (IL-10). Nanoparticles (Poly-(lactic acid)-Polyethylene glycol (PLA-PEG)-IL-10 and PLA-PEG-Phosphate buffered saline (PBS)) were diluted in sterile deionized water and scanned in cuvettes using Malvern Nano-ZS for zeta-sizing (**A**,**B**) and zeta-potential (**C**,**D**). Triplicate readings were obtained for each nanoparticle sample.

As particle size plays an important role in determining the level of cellular and tissue uptake, we employed zeta-sizing, which revealed that both PLA-PEG-PBS and PLA-PEG-IL-10 were within the 200 nm range (Table 1; Figure 2A,B). The significance of the stability of PLA-PEG-PBS and PLA-PEG-IL-10 was also assessed by zeta-potential studies, which respectively disclosed −12.9 mV and −14.2 mV surface charges (Table 1; Figure 2C,D). Moreover, both PLA-PEG-PBS and PLA-PEG-IL-10 were uniform in size, as revealed by their PDI values of 0.225 and 0.256, respectively. These findings demonstrate that the encapsulation process had little effect on changing the properties and size of the nanoparticles, which are essential for maintaining the integrity and delivery potential of the nanoparticles. 

#### 3.3.2. Spectrometry, Differential Scanning Calorimetry (DSC), and In Vitro Release of Encapsulated IL-10

**Figure 3:**
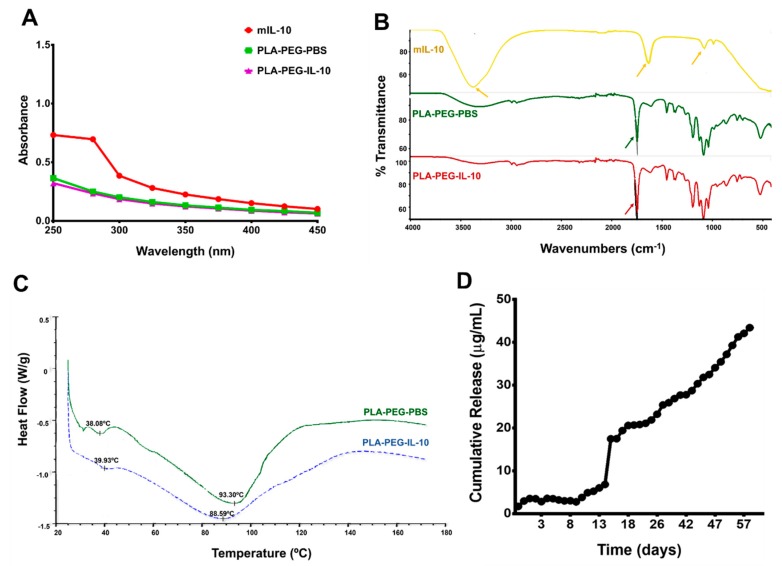
Physiostructural characterization and release of nanoparticles. Nanoparticles and IL-10 were dissolved in DNAse-RNAse free water and analyzed by spectroscopy (**A**). FT-IR spectra were employed to show the various functional groups and differences in the absorption spectra for nanoparticles. The FT-IR spectrum was recorded with 64 scans/sample ranging from 4000 to 1000 cm^−1^ and a resolution of 4 cm^−1^ at ambient temperature (**B**). Thermal stability of IL-10 encapsulated within PLA-PEG nanoparticles was determined by placing nanoparticles in an aluminum pan, sealing it, and then heating it at the rate of 20 °C per min from 30 to 120 °C under nitrogen, before cooling it from 120 to 30 °C at the same rate, using a differential scanning calorimeter. Shown are peaks for PLA-PEG-PBS (green) and PLA-PEG-IL-10 (blue), respectively (**C**). The in vitro release of IL-10 from PLA-PEG nanoparticles was determined by placing nanoparticles in sterile PBS. At each time-point, supernatants were collected and the IL-10 content was measured spectrophotometrically. Shown is the average cumulative release of IL-10 over a period of 60 days, and the experiment was performed three times (**D**). DNA = deoxyribonucleic acid, IL-10 = Interleukin-10, FT-IR = Fourier Transform-Infrared, PBS = Phosphate-buffered saline, PLA-PEG = Poly (lactic acid)-Poly (ethylene glycol), RNA = ribonucleic acid, UV = Ultra-Violet.

To ascertain the full encapsulation of IL-10, we conducted UV-Vis studies using naked IL-10 (positive) and PLA-PEG-PBS (negative) as controls. Our result reported in Figure 3A shows a peak absorbance at a 280 nm wavelength (UV visible region for protein) (red) for IL-10 alone, indicating the presence of IL-10 in the sample solution. However, PLA-PEG-IL-10 nanoparticles exhibited no peak absorbance at the same wavelength, indicating successful IL-10 encapsulation within PLA-PEG nanoparticles (purple). A similar absorbance pattern was observed for PLA-PEG-PBS (green). The UV visible spectra study confirmed that IL-10 was completely encapsulated and not absorbed on the surface of nanoparticles.

We then employed FT-IR to identify variations in chemical functional groups within naked IL-10, PLA-PEG-IL-10, and PLA-PEG-PBS for further validation of the successful encapsulation of IL-10. In contrast to the bare monkey IL-10, the positions of some of the peaks showed differences in appearance between the encapsulated nanoparticles (Figure 3B). We observed unique peak shifts at wavelengths of 3383.81, 1635.16, and 1080.03 cm^−1^ for IL-10, which were absent in PLA-PEG-IL-10 and PLA-PEG-PBS spectra. Appearances of typical characteristic peaks for -C-H stretching (at 3200 cm^−1^), multiple peaks at C-O stretching (at 1452 cm^−1^), -C(O)-O-C-(ester C-O) group formation (at 1342 cm^−1^) [not labelled], and the presence of the ester carbonyl (CO) group (at 1746 cm^−1^) were observed within both PLA-PEG nanoparticles, as previously reported [25,38]. This indicates the successful encapsulation of IL-10 safely within the PLA-PEG formulation, which further protected the IL-10 load from escaping the nanoparticles. 

Thermal stability evaluations were performed to study the physical state of encapsulated IL-10, which could affect its in vitro release pattern. The DSC thermograms of PLA-PEG- PBS (control) and PLA-PEG-IL-10 are depicted in Figure 3C. The melting temperature (Tm) of PEG is ~40 °C. We observed that the melting temperature of PLA shifted from a typical value of 150 °C due to the broadening effect indicative of an interaction between the two monomers and lower crystallinity of PLA components. Furthermore, the endothermic melting peaks for PLA-PEG-PBS appeared at 93.3 °C (green) and for PLA-PEG-IL-10, 88.59 °C (blue), suggesting no significant encapsulation effect on the thermal stability of the PLA-PEG-IL-10 nanoparticles.

Finally, the controlled cumulative release of the encapsulated IL-10 was investigated for up to 60 days. The cumulative release of encapsulated-IL-10 was one of a biphasic release pattern (Figure 3D), where phase one was rapid and lasted up to day 15, followed by a gradual release over a 60-day period for up to a 70% total release. The sustained slow in vitro release of the IL-10 up to day 60 is an attractive attribute because with such a pattern, a minimum dose of IL-10 will potentially be released at the in vivo target sites.

### 3.4. In Vitro Bioactivity of PLA-PEG-IL-10

**Figure 4:**
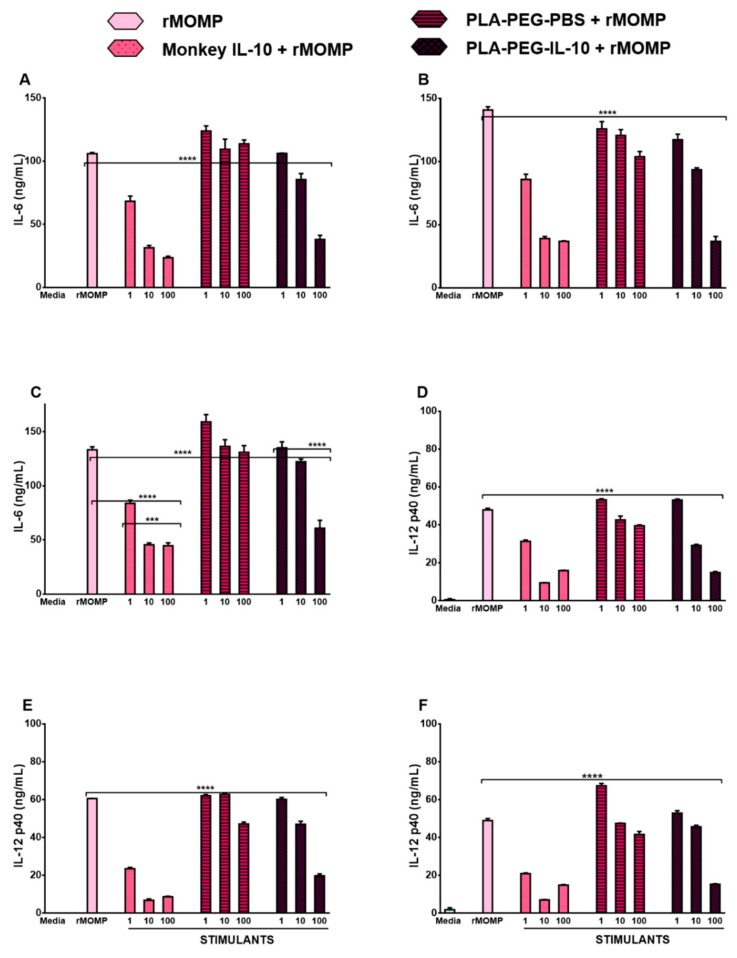
Dose- and time-dependent studies showing down-regulation of IL-6 and IL-12p40 by encapsulated and naked IL-10 in mouse J774 macrophages. Macrophages were stimulated with rMOMP and incubated for over a 72 h period with different concentrations of PLA-PEG-IL-10 (1 to 100 ng/mL) and PLA-PEG-PBS (an equivalent nanoparticle weight). At each time-point, namely 24, 48, and 72 h, cytokines were quantified in cell-free supernatants by specific cytokine ELISAs for IL-6 (**A**–**C**) and IL-12 p40 (**D**–**F**), respectively. *p* values were calculated by the use of one-way ANOVA, followed by Turkey’s Post-test using GraphPad Prism 6 Software. Statistical significance was established and *p* values < 0.05 were considered as statistically significant. *** *p* < 0.001, and **** *p* < 0.0001. Each bar represents the mean ± SD of samples run in triplicate. ELISA = enzyme-linked immunosorbent assay, IL-6 = Interleukin-6, IL-10 = Interleukin-10, IL-12 p40 = Interleukin-12 p40, PBS = Phosphate-buffered saline, PLA-PEG = Poly (lactic acid)-Poly (ethylene glycol), rMOMP = recombinant major outer membrane protein.

The highly immunogenic MOMP of *Chlamydia* contributes to its inflammation by stimulating macrophages to secrete pro-inflammatory cytokines that play a pivotal role in the pathogenesis of the disease [25]. The successful encapsulation, physio-structural, and in vitro release of encapsulated-IL-10 warranted testing its functional anti-inflammatory effects in modulating inflammatory cytokines triggered in mouse J774, in response to rMOMP stimulation. Accordingly, we performed dose-response and time-kinetics studies to assess the cytokine release profile of macrophages after their exposure to rMOMP in the presence and absence of PLA-PEG-IL-10 (1–100 ng/mL) as compared to bare monkey IL-10 (1–100 ng/mL). Herein, we used IL-6 and IL-12 p40 as markers of inflammation to determine the anti-inflammatory effect of PLA-PEG-IL-10 on its secretion from macrophages stimulated with rMOMP for 24–72 h. Stimulation of macrophages with rMOMP resulted in dose-dependent increases in the production of IL-6 (Figure 4A–C) and IL-12p40 (Figure 4D–F) that were further enhanced with each time-point. Of the upmost importance to our study was that our data shows that all concentrations of monkey IL-10 alone (1–100 ng/mL) and encapsulated-IL-10 (1–100 ng/mL) reduced the levels of IL-6 and IL-12 p40 in mouse J774 macrophages post 24–72 h treatment as compared to rMOMP (positive control) and PLA-PEG-PBS concentrations (negative control) (Figure 4A–F). Interestingly, we also observed that high concentrations of PLA-PEG-PBS caused the minimal inhibition of IL-6 and IL-12p40, suggesting that PLA-PEG does possess some immune potentiating abilities that enhance the therapeutic effect of IL-10. Furthermore, the inhibition effect of IL-10 was maintained over time, where the most effective inhibition was observed at 72 h, confirming the slow, accumulated release of encapsulated IL-10 during interaction of the nanoparticles with macrophages for the enhanced suppression of pro-inflammatory responses (Figure 4C,F). Overall, this data provides compelling evidence that encapsulated IL-10 is functional and maintains its anti-inflammatory properties in eukaryotic cells.

### 3.5. Differential SOCS1 and SOCS3 Expressions in Response to the Stimulation of Macrophages with rMOMP and Encapsulated IL-10

**Figure 5:**
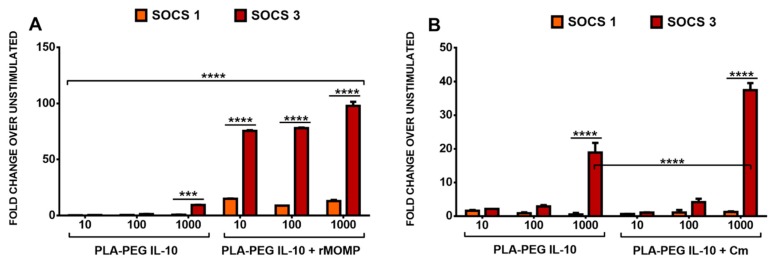
Differential transcriptional expression of SOCS1 and SOCS3 induced by stimulated macrophages. Mouse J774 macrophages (3 × 10^6^ cells/mL) were co-stimulated with various concentrations of PLA-PEG-IL-10 or PLA-PEG-PBS with rMOMP (10 µg/mL) or Live Cm (MOI 2). RNA was collected from cultures at 24 h and analyzed by RT-PCR to determine SOCS1 and SOCS3 mRNA transcript levels. For RT-PCR and quantitative real-time PCR, all values were normalized with respect to GAPDH mRNA levels. Results are presented as the fold increase over the control (i.e., the level in unstimulated cells). The results shown are the means ± standard deviations of three separate experiments (**A**-**B**). Data were analyzed by two-way ANOVA, followed by Tukey’s post-hoc test using GraphPad Prism 6 software. *** *p* < 0.001, and **** *p* < 0.0001. ANOVA = analysis of variance, GAPDH = glyceraldehyde 3-phosphate dehydrogenase, IL-10 = interleukin-10, mRNA = messenger ribonucleic acid, rMOMP = recombinant major outer membrane protein, RT-PCR = real time polymerase chain reaction, SOCS = suppressor of cytokine signaling.

We demonstrated above (Supplementary Data Figure S2) that rMOMP of Cm can induce SOCS1 and SOCS3 expression, which may play a role in controlling inflammation. Additionally, we showed that the inverse induction of both SOCS1 and SOCS3 by IL-10 alone and combined with rMOMP inferred that SOCS might be a part of the IL-10-mediated anti-inflammatory properties in chlamydial-stimulated macrophages. This held promise for the induction of SOCS by encapsulated IL-10 in our present study. Our data demonstrated that PLA-PEG IL-10 at all concentrations studied induced low SOCS1 and increased SOCS3 expression. Our results further revealed that the differential reciprocal expressions of SOCS1 and SOCS3 were maintained with added rMOMP or Cm, further validating that the slow release of IL-10 from our PLA-PEG delivery vehicles is still efficient at regulating SOCS1 and SOCS3 in chlamydial macrophages (Figure 5A,B). This study confirmed a direct connection between the IL-10 inhibitory effect and the induction of SOCS1 and SOCS3 gene transcripts in macrophages by co-stimulation. 

## 4. Discussion and Conclusions

IL-10 is a pleotropic anti-inflammatory cytokine as it regulates the expression of multiple inflammatory mediators elicited from a variety of cell types [39]. Its regulatory effect has been proven against bacterial inflammation [27], including *Chlamydia* [4] and other inflammatory diseases [40]. However, the effective therapeutic potential of IL-10 is limited due to its very short biological half-life, which necessitates frequent administrations in biomedical applications. The use of biodegradable nanoparticles as delivery vehicles and immune-potentiators is now playing an increasing role in therapeutic studies. 

Polymeric nanoparticles formulated from biodegradable polymers are being widely explored as immune potentiators and more importantly, viable carriers for the site-specific delivery of vaccines, genes, drugs, and other biomolecules (such as proteins) in the body [17]. Specifically, PLA and PEG polymers are being widely investigated for developing nano-encapsulating therapeutic materials in sustained drug release applications due to some inherent advantages [41,42]. Our results presented in this study revealed that encapsulated IL-10 in the polymeric biodegradable PLA-PEG nanoparticles did prevent the rapid degradation of IL-10. The PLA-PEG nanoparticles prolonged the release of encapsulated IL-10 up until 60 days, while maintaining its anti-inflammatory properties in vitro. 

PLA-PEG nanoparticles have been widely used for enhancing the drug loading of hydrophobic drugs, reducing the burst effect, avoiding phagocytosis by macrophages, enhancing the stability of the drug, and improving the bioavailability [42,43]. Manickavasagam et al. engineered PLA-PEG polymersomes loaded with the drug Simvastatin, which amplified the anti-inflammatory effects in activated microglia, hereby reducing neuroinflammation [44]. PEG was also used as an efficient delivery system for the anti-cancer drug paclitaxel, hereby enhancing the inhibition of tumor growth in in vivo studies [45]. Similarly, PEG was successfully employed for the site-specific delivery of drugs to treat various cancers [46,47,48,49]. 

Further, PLA-PEG has been shown to exhibit targeted drug delivery and sustained, slow release immune-potentiating properties up to several weeks, which is an attractive property for our research studies [50]. Ishihara et al. showed that a PEG-PLA and PLA nanoparticle mixture loaded with betamethasone disodium phosphate (BP) not only enhanced the anti-inflammatory effect of the drug, but also preferentially localized in the lesions of inflammatory mice, where it subsequently and slowly released BP. Similarly, Hami et al. demonstrated that doxorubicin conjugated PLA-PEG-Folate-based polymeric micelles for tumor-targeted delivery [51]. A study by Rafat et al. showed that PEG-PLA encapsulated transcription-enhanced green fluorescent protein fusion (Tat-EGFP) localized within the photoreceptor layer of the retina and persisted for at least 9 weeks, with no observed toxicity to retinal cells [52]. PLA-PEG was also used as a carrier for the safe and efficient sustained release of recombinant human Growth hormone (rhGH) for therapeutic studies [53]. Of significance to our study, therefore, was that we propose that this extended controlled release of IL-10 from PLA-PEG-encapsulated-IL-10 may be beneficial to potentially limiting the administration of high doses of IL-10, which has been a major drawback in clinical efforts. In an IL-10 phase 2 trial for psoriasis treatment, patients received 20 µg/kg three times per week for 7 weeks and while the results were favorable and safe [54], a decreased dosage and less frequent hospital visits may offer considerable relief to some patients. Other nanomaterials, such as dextrin nanogels, have been used to encapsulate IL-10, which provided a successful slow release of IL-10 both in vitro [50] and in vivo [55], but their results revealed the partial release of IL-10 up to only 4 h. Essentially, our study has thus demonstrated a stronger interaction between IL-10 and PLA-PEG nanoparticles, showing a slow release pattern of IL-10 from PLA-PEG up to 60 days. Of major significance, our observed 70% release of encapsulated IL-10 over the 60-day period makes the PLA-PEG delivery system highly attractive for the delivery of IL-10 in clinical applications. Additionally, we show, to our knowledge, the first report of encapsulating IL-10 in a combined PLA-PEG nanomaterial followed by characterization studies and bioactivity in vitro to demonstrate the efficacious inhibition of inflammatory responses elicited by chlamydial rMOMP in macrophages. 

Successful encapsulation of proteins within polymeric nanoparticles is dependent on many factors, including the molecular weight, a charge of the nanomaterials, the size of the peptide, and the co-emulsifier added to the primary emulsion [56]. Herein, we encapsulated IL-10 in PLA-PEG nanoparticles and achieved a 77% encapsulation efficiency. The loss of encapsulation efficiency of IL-10 could be attributed to the loss of nanoparticles due to the multiple sonication steps and lengthy lyophilization required to retrieve solid nanoparticles at the end of the encapsulation process. Our PLA-PEG IL-10 particle size was found to be in the range of ~238 nm, exhibiting the proper size to facilitate their rapid uptake by immune cells, such as macrophages. Such a size difference between the cells and nanoparticles is advantageous for minimal interference within the cell and provides great potential for more prolonged therapy. 

The physical state and stability of nanoparticles can be considered important parameters for the efficient delivery and maintenance of their native properties before entering cells. In the present study, the encapsulated IL-10 was determined to be very stable, with endothermic peaks up to 89 °C, as shown by differential scanning calorimetry analysis, ensuring its stability during in vitro and future in vivo administration.

Lastly, we have demonstrated that encapsulated IL-10 dose-dependently suppressed IL-6 and IL-12 p40 as induced, in response to the rMOMP stimulation of macrophages Our data showed that IL-10 was still functional as it exerted its anti-inflammatory activity in vitro up to 72 h at all the concentrations examined, suggesting that encapsulated IL-10 bioactivity is functionally active and able to exert its anti-inflammatory effect. Moreover, a primary role of SOCS3, like SOCS1, is to control the duration of cytokine signaling in macrophages, although SOCS1 and SOCS3 are not functionally interchangeable for this response. We showed that the ability of IL-10 to differentially regulate SOCS1 and SOCS3 correlated with the suppression of pro-inflammatory mediators in the chlamydial milieu. Furthermore, the reciprocal regulation of SOCS1 and SOCS3 by IL-10 in macrophages is of interest and warrants studies to explore their role in IL-10 anti-inflammatory effects during chlamydial-induced inflammation. 

Overall, our results clearly demonstrate that the use of PLA-PEG nanoparticles provides an effective delivery system for IL-10 as it prolonged its biological half-life and provided its sustained slow release, while maintaining its functional anti-inflammatory properties in modulating chlamydial inflammatory mediators in vitro. The immunotherapeutic effect of encapsulated IL-10 on inflammatory responses provides proof of concept for its application in bacterial, inflammatory, and autoimmune diseases. 

## Figures and Tables

**Table 1:** Nanoparticle size distribution, zeta-potential, and polydispersity index (PDI) measurements, and encapsulation efficiency.

Nanoparticles	Zeta-Sizer (nm)	Zeta-Potential (mV)	PDI	Encapsulation Efficiency
PLA-PEG-PBS	265.5	−12.9	0.225	-
PLA-PEG-IL-10	238.2	−14.2	0.256	~77%

## Supplementary Materials

The following are available online at https://www.mdpi.com/2079-4991/9/8/1074/s1: Figure S1: Monkey recombinant IL-10 exhibits anti-inflammatory properties in mouse macrophages by down-regulating the translational release of IL-6; Figure S2: *Chlamydia* rMOMP activates the mRNA gene transcripts of the cytokine signaling molecules (SOCS1 and SOCS3) and the transcription factors (STAT1 and STAT3), either alone or combined with mouse IL-10 in macrophages.
