# Supplementary material for: Prolonged Release and Functionality of Interleukin-10 Encapsulated within PLA-PEG Nanoparticles
Source: Nanomaterials (Basel). 2019 Jul 26;9(8):1074. doi: 10.3390/nano9081074 (PMC6723354; doi:10.3390/nano9081074)
Supplement: Supplementary file 1 [file nanomaterials-09-01074-s001.pdf]

Supplementary Data:

Prolonged Release and Functionality of Interleukin-10 Encapsulated within PLA-PEG Nanoparticles

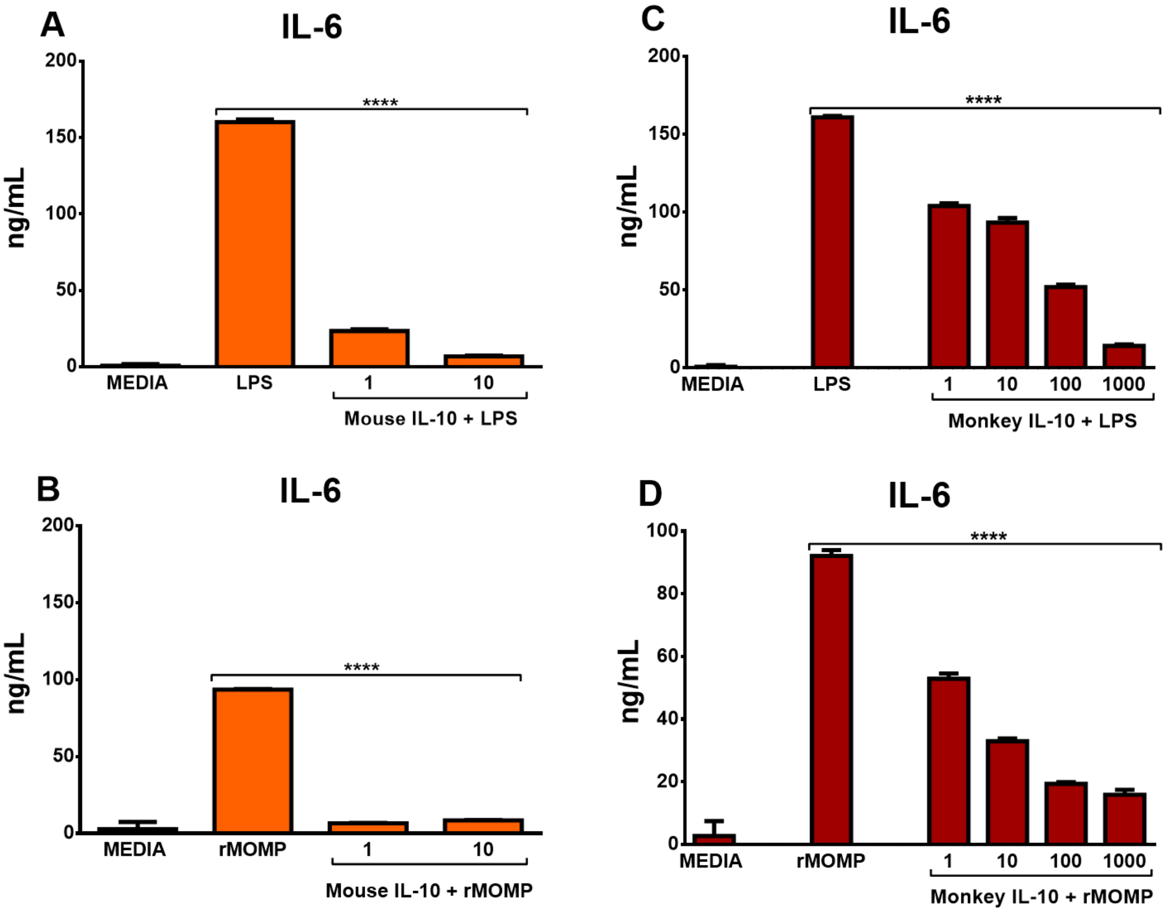

Figure S1. Monkey recombinant IL-10 exhibits anti-inflammatory properties in mouse macrophages by down-regulating the translational release of IL-6. Mouse J774 macrophages (3×10^6 cells/mL) were seeded in 24-well plates and stimulated with LPS (1 μg/mL) or rMOMP (10 μg/mL) in the presence and absence of varying dosages of mouse (1 and 10 ng/mL) and monkey (1, 10, 100 and 1000 ng/mL) recombinant IL-10. Cell-free culture supernatants were collected after 24 h to quantify IL-6 (**A-D**) by specific ELISA. IL-6 levels are shown in nanograms/milliliter (ng/mL). An asterisk indicates significant differences between macrophages stimulated with LPS or rMOMP and those with added recombinant IL-10. *P* values were calculated by the use of one-way ANOVA followed by Turkey's Post-test using GraphPad Prism 6 Software. Statistical significance was established and *P* values < 0.05 were considered as statistically significant (*$P < 0.05$; **$P < 0.01$, ***$P < 0.001$ and ****$P < 0.0001$). Each bar represents the mean ± SD) of samples run in triplicates.

Abbreviations: analysis of variance, ANOVA; ELISA, enzyme-linked immunosorbent assay; IL-10, interleukin-10; lipopolysaccharide, LPS; rMOMP, recombinant major outer membrane protein; SD, standard deviation.

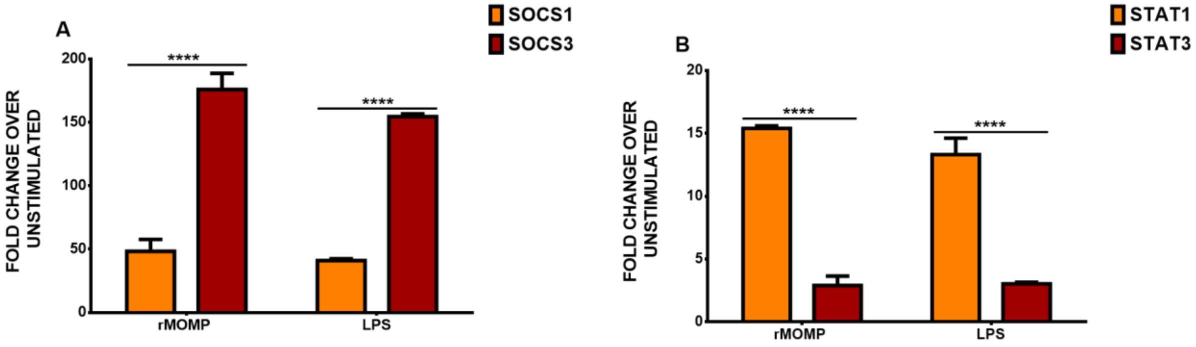

Figure S2. ***Chlamydia*** **rMOMP activates the mRNA gene transcripts of the cytokine signaling molecules (SOCS1 and SOCS3) and the transcription factors (STAT1 and STAT3) either alone or combined with mouse IL-10 in macrophages.** Mouse J774 macrophages were exposed to rMOMP (10 μg/mL) and 24 h post-stimulation RNA samples were collected for quantification of the mRNA gene transcripts for SOCS1 and SOCS3 **(A)** and STAT1 and STAT3 **(B)** using TaqMan qRT-PCR. Mouse J774 macrophages were exposed to rMOMP (10 μg/mL) in the presence and absence of mouse IL-10 (10 ng/mL) for 0-24 h. All values were normalized with respect to the mRNA levels of the "housekeeping" gene that codes for GAPDH. Results are presented as fold increase over the control (i.e., the level in unstimulated cells. *P* values were calculated by the use of one-way ANOVA followed by Turkey's Post-test using GraphPad Prism 6 Software. Statistical significance was established and *P* values < 0.05 were considered as statistically significant (*$P < 0.05$; **$P < 0.01$, ***$P < 0.001$ and ****$P < 0.0001$). Each bar represents the mean ± SD) of samples run in triplicates.

Abbreviations: analysis of variance, ANOVA; GAPDH, glyceraldehyde 3-phosphate dehydrogenase; mRNA, messenger ribonucleic acid; rMOMP, recombinant major outer membrane protein; RT-PCR, real time polymerase chain reaction; SOCS, suppressor of cytokine signaling.
